# Supplementary material for: Voice Over Body? Older Adults’ Reactions to Robot and Voice Assistant Facilitators of Group Conversation
Source: Int J Soc Robot. 2022 Nov 11;15(2):143–63. doi: 10.1007/s12369-022-00925-7 (PMC9651097; doi:10.1007/s12369-022-00925-7)
Supplement: Supplementary file 1 — Supplementary Material 1 [file 12369_2022_925_MOESM1_ESM.docx]

OR1. Talkativeness Questionnaire (EN)

Article title: Voice over body? Older adults’ reactions to robot and voice assistant facilitators of group conversation

Journal: International Journal of Social Robotics

Authors: [authors removed for review]^1^*

^1^[affiliation of corresponding author removed for review]

*Corresponding author: [email address of corresponding author removed for review]

# Questionnaire About Everyday Conversation

For each of the following, please circle the answer that most closely matches you.

To start, we would like to know about your conversation style **in general**.

C1. I’m basically a talkative person.

　　Yes, I agree Neither agree nor disagree No, I don't agree

Next, we would like to ask you about your conversation style in **specific situations**.

C2. Rate the amount of talking you do during a conversation with a good friend.

　　A lot A fair amount Some A little

C3. Rate the amount of talking you do during a conversation with someone you haven’t had much contact with.

　　A lot A fair amount Some A little

This is the end of the survey. Thank you very much for your cooperation.
