# Supplementary material for: Voice Over Body? Older Adults’ Reactions to Robot and Voice Assistant Facilitators of Group Conversation
Source: Int J Soc Robot. 2022 Nov 11;15(2):143–63. doi: 10.1007/s12369-022-00925-7 (PMC9651097; doi:10.1007/s12369-022-00925-7)
Supplement: Supplementary file 2 — Supplementary Material 2 [file 12369_2022_925_MOESM2_ESM.docx]

OR2. Talkativeness Questionnaire (JP)

Article title: Voice over body? Older adults’ reactions to robot and voice assistant facilitators of group conversation

Journal: International Journal of Social Robotics

Authors: [authors removed for review]^1^*

^1^[affiliation of corresponding author removed for review]

*Corresponding author: [email address of corresponding author removed for review]

# 普段の会話に関するアンケート

次の質問について、該当するものを選んで〇をつけてください。

まずは、ご自身の**会話一般**についての質問です。

C1. 私は基本的にはおしゃべりな方だ。

　　あてはまる　　どちらともいえない　　あてはまらない

次に、**具体的な場面**での会話についておたずねします。

C2. 仲の良い人との会話中にあなたが話す量を評価してください。

　　発言が多い　　やや発言が多い　　やや発言が少ない　　発言が少ない

C3.あまり関わったことがない人との会話中にあなたが話す量を評価してください。

　　発言が多い　　やや発言が多い　　やや発言が少ない　　発言が少ない

以上でアンケートは終了になります。ご協力頂きどうもありがとうございました。
