# Supplementary material for: Voice Over Body? Older Adults’ Reactions to Robot and Voice Assistant Facilitators of Group Conversation
Source: Int J Soc Robot. 2022 Nov 11;15(2):143–63. doi: 10.1007/s12369-022-00925-7 (PMC9651097; doi:10.1007/s12369-022-00925-7)
Supplement: Supplementary file 3 — Supplementary Material 3 [file 12369_2022_925_MOESM3_ESM.docx]

OR3. Embodiment Questionnaire (EN)

Article title: Voice over body? Older adults’ reactions to robot and voice assistant facilitators of group conversation

Journal: International Journal of Social Robotics

Authors: [authors removed for review]^1^*

^1^[affiliation of corresponding author removed for review]

*Corresponding author: [email address of corresponding author removed for review]

# Questionnaire about Robot Facilitation

The purpose of this questionnaire is to gather your opinions on and experience with the robot facilitator. Be sure to read and answer all items carefully.

| I. For each of the following questions, please circle the answer that most closely matches your perspective. **Be sure to answer all items.** | | | | | | |
| --- | --- | --- | --- | --- | --- | --- |
|  | | Disagree | Somewhat disagree | Neither | Somewhat agree | Agree |
| B1. | The robot’s facilitation was easy to understand | 1 | 2 | 3 | 4 | 5 |
| B2. | The robot’s facilitation was fun. | 1 | 2 | 3 | 4 | 5 |
| B3. | I was confused by the robot’s facilitation. | 1 | 2 | 3 | 4 | 5 |
| B4. | I got used to the robot’s facilitation. | 1 | 2 | 3 | 4 | 5 |
| B5. | I was able to hear the robot’s voice well. | 1 | 2 | 3 | 4 | 5 |
| B6. | The timing of the robot’s speech was appropriate. | 1 | 2 | 3 | 4 | 5 |
| B7. | The robot spoke at the right speed. | 1 | 2 | 3 | 4 | 5 |
| B8. | The robot’s appearance was appropriate. | 1 | 2 | 3 | 4 | 5 |
| B9. | The robot’s appearance made me feel relaxed. | 1 | 2 | 3 | 4 | 5 |
| I Continued. For each of the following questions, please circle the answer that most closely matches your perspective. **Be sure to answer all items.** | | | | | | |
|  | | Disagree | Somewhat disagree | Neither | Somewhat agree | Agree |
| B10. | I accepted the robot stopping me from speaking too much. | 1 | 2 | 3 | 4 | 5 |
| B11. | The robot helped me to speak when I spoke too little. | 1 | 2 | 3 | 4 | 5 |
| B13. | The robot is better than a person at interrupting speech.  → Reason：（　　　　　　　　　　　　　　　　　） | 1 | 2 | 3 | 4 | 5 |
| B14. | The robot is better than a person at encouraging speech.  → Reason：（　　　　　　　　　　　　　　　　　） | 1 | 2 | 3 | 4 | 5 |
| B15. | The robot is better at facilitation than a person.  → Reason：（　　　　　　　　　　　　　　　　　） | 1 | 2 | 3 | 4 | 5 |
| U1. | The size of the robot was appropriate. | 1 | 2 | 3 | 4 | 5 |
| U2. | It was fun to interact with the robot. | 1 | 2 | 3 | 4 | 5 |
| U3. | Talking with this robot feels like talking with a person. | 1 | 2 | 3 | 4 | 5 |
| U4. | I can interact with the robot like I interact with people. | 1 | 2 | 3 | 4 | 5 |
| U5. | I liked that the robot looked similar to a human. | 1 | 2 | 3 | 4 | 5 |
| U6. | I perceived the robot as an autonomous actor. | 1 | 2 | 3 | 4 | 5 |
| U7. | I liked that the robot has human-like features, e.g., face, eyes, etc. | 1 | 2 | 3 | 4 | 5 |
| U8. | I felt good when interacting with the robot. | 1 | 2 | 3 | 4 | 5 |
| U9. | I liked that I was physically co-located with the robot. | 1 | 2 | 3 | 4 | 5 |
| U10. | I thought that the robot was intelligent. | 1 | 2 | 3 | 4 | 5 |
| U11. | I liked the design of the robot. | 1 | 2 | 3 | 4 | 5 |
| U12. | I enjoyed being talked to by the robot. | 1 | 2 | 3 | 4 | 5 |
| U13. | The robot could become a companion for me. | 1 | 2 | 3 | 4 | 5 |
| U14. | I felt secure when interacting with the robot. | 1 | 2 | 3 | 4 | 5 |
| I Continued. For each of the following questions, please circle the answer that most closely matches your perspective. **Be sure to answer all items.** | | | | | | |
|  | | Disagree | Somewhat disagree | Neither | Somewhat agree | Agree |
| U15. | I felt afraid of the robot. | 1 | 2 | 3 | 4 | 5 |
| U16. | I felt that the robot was not dangerous. | 1 | 2 | 3 | 4 | 5 |
| SB1. | I want to use this robot when talking in groups. | 1 | 2 | 3 | 4 | 5 |
| SB5. | I felt that the various functions of this robot were well organized, e.g., its movements and ability to facilitate. | 1 | 2 | 3 | 4 | 5 |
| SB8. | The robot’s facilitation was not efficient. | 1 | 2 | 3 | 4 | 5 |

B01. Please tell us your impressions about the robot-facilitated Coimagination session that you experienced.

B02. Please tell us the good points and points for improvement.

BO3. Please tell us if there are any additions you would like for the robot.

II. For each of the following questions, please circle the answer that most closely matches your perspective. **Be sure to answer all items.** If you don’t remember, please circle the “I can’t recall” option.

A1. What color is the robot's **head**? (See the figure in question A3 below)

　　Peach Red Orange Yellow Green Blue Purple Gray Black White I can’t recall

A2. What color is the robot's **body**? (See the figure in question A3 below)

　　Peach Red Orange Yellow Green Blue Purple Gray Black White I can’t recall

**7. I can’t recall.**

A3. Please circle the facial expression that is most similar to the robot’s facial expression. If you don’t know, please circle that option.

A4. How **confident** are you about your answer to question A3 above?

Not at all　　A little　　Confident　　Very confident

A5. Did you notice anything else about the robot over the course of the session?

This is the end of the questionnaire. Thank you for your cooperation.
