# Supplementary material for: Voice Over Body? Older Adults’ Reactions to Robot and Voice Assistant Facilitators of Group Conversation
Source: Int J Soc Robot. 2022 Nov 11;15(2):143–63. doi: 10.1007/s12369-022-00925-7 (PMC9651097; doi:10.1007/s12369-022-00925-7)
Supplement: Supplementary file 4 — Supplementary Material 4 [file 12369_2022_925_MOESM4_ESM.docx]

OR4. Embodiment Questionnaire (JP)

Article title: Voice over body? Older adults’ reactions to robot and voice assistant facilitators of group conversation

Journal: International Journal of Social Robotics

Authors: [authors removed for review]^1^*

^1^[affiliation of corresponding author removed for review]

*Corresponding author: [email address of corresponding author removed for review]

# ロボットの司会に関してのアンケート

このアンケートでは、ロボットによる司会に関するあなたのご意見を様々な観点からお尋ねしています。必ず、すべての項目を慎重に読んで回答してください。

| I.次の質問のそれぞれの内容について、5段階の中で、自分に最も該当するものを選んで〇をつけてください。必ず、**すべての項目に回答**してください。 | | | | | | |
| --- | --- | --- | --- | --- | --- | --- |
|  | | 当てはまらない | あまり当てはまらない | どちらでもない | やや当てはまる | 当てはまる |
| B1. | このロボットの司会進行は分かりやすかった。 | 1 | 2 | 3 | 4 | 5 |
| B2. | このロボットが司会進行するのは面白かった。 | 1 | 2 | 3 | 4 | 5 |
| B3. | このロボットの司会進行に戸惑った。 | 1 | 2 | 3 | 4 | 5 |
| B4. | このロボットの司会進行に慣れた。 | 1 | 2 | 3 | 4 | 5 |
| B5. | このロボットの声はよく聞こえた。 | 1 | 2 | 3 | 4 | 5 |
| B6. | このロボットが声を出すタイミングは適切だった。 | 1 | 2 | 3 | 4 | 5 |
| I続き.次の質問のそれぞれの内容について、5段階の中で、自分に最も該当するものを選んで〇をつけてください。必ず、**すべての項目に回答**してください。 | | | | | | |
|  | | 当てはまらない | あまり当てはまらない | どちらでもない | やや当てはまる | 当てはまる |
| B7. | このロボットが話す速さは適切だった。 | 1 | 2 | 3 | 4 | 5 |
| B8. | このロボットの外観は適切だった。 | 1 | 2 | 3 | 4 | 5 |
| B9. | このロボットの見た目で気持ちが和んだ。 | 1 | 2 | 3 | 4 | 5 |
| B10. | 私の発言が多い時に発言を止めるのはこのロボットでもよい。 | 1 | 2 | 3 | 4 | 5 |
| B11. | 私の発言が少ない時に発言を促すのはこのロボットでもよい。 | 1 | 2 | 3 | 4 | 5 |
| B13. | 発言を止めるのは人間よりこのロボットがよい。  →その理由：（　　　　　　　　　　　　　　　　　） | 1 | 2 | 3 | 4 | 5 |
| B14. | 発言を促すのは人間よりこのロボットがよい。  →その理由：（　　　　　　　　　　　　　　　　　） | 1 | 2 | 3 | 4 | 5 |
| B15. | 司会進行は人間よりこのロボットがよい。  →その理由：（　　　　　　　　　　　　　　　　　） | 1 | 2 | 3 | 4 | 5 |
| U1. | このロボットの大きさは適切だった。 | 1 | 2 | 3 | 4 | 5 |
| U2. | このロボットと接するのは楽しかった。 | 1 | 2 | 3 | 4 | 5 |
| U3. | このロボットと喋るうちに人間と喋っているかのように 感じた。 | 1 | 2 | 3 | 4 | 5 |
| U4. | 人間と接するのと同じようにこのロボットとも接することができる。 | 1 | 2 | 3 | 4 | 5 |
| U5. | このロボットの見た目が人間らしいところがよかった。 | 1 | 2 | 3 | 4 | 5 |
| U6. | このロボットは自律した主体だと感じた。 | 1 | 2 | 3 | 4 | 5 |
| I続き.次の質問のそれぞれの内容について、5段階の中で、自分に最も該当するものを選んで〇をつけてください。必ず、**すべての項目に回答**してください。 | | | | | | |
|  | | 当てはまらない | あまり当てはまらない | どちらでもない | やや当てはまる | 当てはまる |
| U7. | このロボットの人間らしい特徴が好きだ。例えば、 顔や耳や目など。 | 1 | 2 | 3 | 4 | 5 |
| U8. | このロボットと接しているときに気分が良かった。 | 1 | 2 | 3 | 4 | 5 |
| U9. | このロボットが身近にいる感じがして良かった。 | 1 | 2 | 3 | 4 | 5 |
| U10. | このロボットには知能があると感じた。 | 1 | 2 | 3 | 4 | 5 |
| U11. | このロボットのデザインが好きだ。 | 1 | 2 | 3 | 4 | 5 |
| U12. | このロボットに話しかけられたのが楽しかった。 | 1 | 2 | 3 | 4 | 5 |
| U13. | このロボットと仲良くなれるかもしれない。 | 1 | 2 | 3 | 4 | 5 |
| U14. | このロボットと接するうちに安心感を覚えた。 | 1 | 2 | 3 | 4 | 5 |
| U15. | このロボットは怖いと思った。 | 1 | 2 | 3 | 4 | 5 |
| U16. | このロボットは危険ではないと感じた。 | 1 | 2 | 3 | 4 | 5 |
| SB1. | グループで会話するときにこのロボットを使いたいと思う。 | 1 | 2 | 3 | 4 | 5 |
| SB5. | このロボットにあるさまざまな機能がよくまとまっていると感じた。例えば、ロボットの動きや司会進行など。 | 1 | 2 | 3 | 4 | 5 |
| SB8. | このロボットの司会進行は効率的ではなかった。 | 1 | 2 | 3 | 4 | 5 |

B01.ロボットが司会進行する共想法に参加した感想を教えてください。

B02.ロボットによる司会進行のよかった点、改善点を教えてください。

BO3.ロボットに追加してほしい発言があれば教えてください。

II. 次の質問について、該当するものを選んで〇をつけてください。必ず、**すべての項目に回答**してください。わからないときは「覚えていない」を選んでください。

A1. ロボットの**頭の色**は何色ですか？（下の質問A3にある図を見てください）

　　桃　　赤　　橙　　黄　　緑　　青　　紫　　灰　　黒　　白　　覚えていない

A2.ロボットの**体の色**は何色ですか？（下の質問A3にある図を見てください）

　　桃　　赤　　橙　　黄　　緑　　青　　紫　　灰　　黒　　白　　覚えていない

A3.あなたが**最も似ている**と思うロボットの表情はどれですか。あてはまる番号を丸で囲んで下さい。

A4.上のロボットの顔を選んでいただいた質問A3について、あなたはどれだけ自分の回答に対して**自信があり**ますか？

全く自信がない　　あまり自信がない　　やや自信がある　　とても自信がある

A5.会話中にロボットに関して、他に何か気づきましたか？

以上でアンケートは終了になります。ご協力頂きどうもありがとうございました。
