# Supplementary material for: Voice Over Body? Older Adults’ Reactions to Robot and Voice Assistant Facilitators of Group Conversation
Source: Int J Soc Robot. 2022 Nov 11;15(2):143–63. doi: 10.1007/s12369-022-00925-7 (PMC9651097; doi:10.1007/s12369-022-00925-7)
Supplement: Supplementary file 5 — Supplementary Material 5 [file 12369_2022_925_MOESM5_ESM.docx]

OR5. Supplementary Tables

Article title: Voice over body? Older adults’ reactions to robot and voice assistant facilitators of group conversation

Journal: International Journal of Social Robotics

Authors: [authors removed for review]^1^*

^1^[affiliation of corresponding author removed for review]

*Corresponding author: [email address of corresponding author removed for review]

Table 1. Measures across studies. Pre: Preliminary study. Main: Main study.

| **Measure** | **Type** | **Method** | **Instrument** | **Data** | **Analysis** | **Used In** |
| --- | --- | --- | --- | --- | --- | --- |
| Usability | Subjective | Questionnaire | SUS | Quantitative self-reports | Statistics | Pre, Main |
| UX | Subjective | Questionnaire | USUS UX | Quantitative self-reports | Statistics | Pre, Main |
| Usability/UX | Subjective | Questionnaire | Custom items | Quantitative self-reports | Statistics | Pre, Main |
| Usability/UX | Subjective | Questionnaire | Open-ended questions | Qualitative comments | Content analysis | Main |
| Usability/UX | Objective | Observation | Written notes | Qualitative notes | Content analysis | Main |
| Talkativeness | Subjective | Pre-Questionnaire | Custom items | Quantitative self-reports | Statistics | Main |
| Awareness-Morphology | Subjective | Questionnaire | Custom items | Mixed | Statistics, thematic analysis | Pre, Main |
| Awareness-Morphology | Objective | Questionnaire | Open-ended question | Qualitative comments | Quantitative coding (noticed or not) | Main |
| Awareness-Morphology | Objective | Metrics | Time (ms/s) | Quantitative | Statistics | Main |
