# Supplementary material for: Voice Over Body? Older Adults’ Reactions to Robot and Voice Assistant Facilitators of Group Conversation
Source: Int J Soc Robot. 2022 Nov 11;15(2):143–63. doi: 10.1007/s12369-022-00925-7 (PMC9651097; doi:10.1007/s12369-022-00925-7)
Supplement: Supplementary file 6 — Supplementary Material 6 [file 12369_2022_925_MOESM6_ESM.docx]

OR6. Supplementary Statistics

Article title: Voice over body? Older adults’ reactions to robot and voice assistant facilitators of group conversation

Journal: International Journal of Social Robotics

Authors: [authors removed for review]^1^*

^1^[affiliation of corresponding author removed for review]

*Corresponding author: [email address of corresponding author removed for review]


Table 1. Descriptive statistics for the preliminary study.

| **Measure** | **Sub-Measure** | **Mean** | **St. Dev.** | **Median** | **IQR** | **Range** | **α** |
| --- | --- | --- | --- | --- | --- | --- | --- |
| Awareness-Morphology | Correctness | 2.0 | 0.8 | 2 | 1 | 0-3 |  |
|  | Confidence | 1.7 | 0.8 | 2 | 2 | 0-3 |  |
|  | PRODUCT | 6.4 | 3.6 | 6 | 12 | 0-16 |  |
| Robot UX | Voice Satisfaction | 1.9 | 0.7 | 2 | 1 | 0-3 |  |
|  | Bono-UX | 41.3 | 8.8 | 40.5 | 13.8 | 0-70 | .94 |
|  | USUS | 40.4 | 8.7 | 41 | 22 | 0-64 | .90 |
|  | SUS | 7.8 | 2.3 | 8 | 5 | 0-12 | .73 |
|  | CONSTRUCT | 91.3 | 19 | 92.5 | 39.25 | 41-120 | .96 |

Table 2. Descriptive statistics for the main study.

| **Measure** | **Sub-Measure** | **Mean** | **St. Dev.** | **Median** | **Int.-Qu.** | **Range** | **α** |
| --- | --- | --- | --- | --- | --- | --- | --- |
| Awareness-Morphology | Correctness | 2.4 | 1.1 | 2 | 2.5 | 0-3 |  |
|  | Confidence | 1.6 | 0.9 | 2 | 1.5 | 0-3 |  |
|  | PRODUCT | 6.5 | 4.2 | 6 | 13 | 2-16 |  |
| Robot UX | Bono-UX | 42.8 | 6.9 | 45 | 14.5 | 0-70 | .78 |
|  | USUS | 44.9 | 10.3 | 44 | 24.5 | 0-64 | .93 |
|  | SUS | 8.7 | 2.1 | 8 | 4 | 0-12 | .58 |
|  | CONSTRUCT | 96.4 | 16.4 | 98 | 38 | 47-126 | .91 |
| Voice UX | Bono-UX | 40.9 | 8.3 | 43 | 16 | 0-70 | .87 |
|  | USUS | 32.1 | 11 | 35 | 30.3 | 0-64 | .93 |
|  | SUS | 7.7 | 2.5 | 8 | 5 | 0-12 | .61 |
|  | CONSTRUCT | 80.6 | 18.2 | 79 | 45.5 | 41-120 | .93 |
