# Supplementary material for: Voice Over Body? Older Adults’ Reactions to Robot and Voice Assistant Facilitators of Group Conversation
Source: Int J Soc Robot. 2022 Nov 11;15(2):143–63. doi: 10.1007/s12369-022-00925-7 (PMC9651097; doi:10.1007/s12369-022-00925-7)
Supplement: Supplementary file 7 — Supplementary Material 7 [file 12369_2022_925_MOESM7_ESM.docx]

OR7. Video

Article title: Voice over body? Older adults’ reactions to robot and voice assistant facilitators of group conversation

Journal: International Journal of Social Robotics

Authors: [authors removed for review]^1^*

^1^[affiliation of corresponding author removed for review]

*Corresponding author: [email address of corresponding author removed for review]

Link to video

Below is an anonymous link to the video, hosted on OSF. We were unable to upload the video to the system due to file size limits that the tech staff were unable to lift. We have uploaded a captions/subtitles file (.srt) with the same name as this document.

<https://osf.io/fqhs9/?view_only=720b06edb2914828bbdd2ee4fa803777>

Duration: 22 sec
File Size: 55.3 mb
Format: mp4
Aspect ratio: 16:9
Size: 1920 × 1080
